# Imaging Predictors of Left Ventricular Functional Recovery after Reperfusion Therapy of ST-Elevation Myocardial Infarction Assessed by Cardiac Magnetic Resonance

**DOI:** 10.3390/jcdd10070294

**Published:** 2023-07-11

**Authors:** Agneta Virbickiene, Tomas Lapinskas, Christoph D. Garlichs, Stephan Mattecka, Radu Tanacli, Wolfgang Ries, Jan Torzewski, Franz Heigl, Christian Pfluecke, Harald Darius, Hueseyin Ince, Peter Nordbeck, Christian Butter, Andreas Schuster, Steffen Mitzner, Olivija Dobiliene, Ahmed Sheriff, Sebastian Kelle

**Affiliations:** 1 Department of Internal Medicine/Cardiology, German Heart Center Berlin, 13353 Berlin, Germany; agneta.virbickiene@gmail.com (A.V.); tomas.lapinskas@lsmuni.lt (T.L.); tanacli@dhzb.de (R.T.); 2 Department of Cardiology, Medical Academy, Lithuanian University of Health Sciences, 44307 Kaunas, Lithuania; olivija.dobiliene@kaunoklinikos.lt; 3 Medical Clinic, DIAKO Flensburg, 24939 Flensburg, Germany; garlichsch@diako.de (C.D.G.); rieswo@diako.de (W.R.); 4 Pentracor GmbH, 16761 Hennigsdorf, Germany; mattecka@pentracor.de (S.M.); sheriff@pentracor.de (A.S.); 5 Department of Cardiology, Charité University Medicine Berlin, 10117 Berlin, Germany; 6 Cardiovascular Center Oberallgäu-Kempten, 87439 Kempten, Germany; jan.torzewski@klinikverbund-allgaeu.de; 7 Medical Care Center Kempten-Allgäu, 87437 Kempten, Germany; heigl@mvz-kempten.de; 8 Christian Pfluecke, Department of Internal Medicine I, Städtisches Klinikum Görlitz, Girbigsdorfer Straße 1-3, 02828 Görlitz, Germany; christian.pfluecke@mailbox.tu-dresden.de; 9 Clinic for Cardiology, Angiology, Nephrology, Intensive Care Medicine, Vivantes Clinic Neukölln, 12351 Berlin, Germany; harald.darius@vivantes.de; 10 Divisions of Cardiology and Nephrology, Department of Internal Medicine, University Medicine Rostock, 18057 Rostock, Germany; hueseyin.ince@med.uni-rostock.de (H.I.); steffen.mitzner@med.uni-rostock.de (S.M.); 11 Department of Internal Medicine I, University Hospital Wuerzburg, 97080 Wuerzburg, Germany; nordbeck_p@ukw.de; 12 Department of Cardiology, University Hospital Heart Centre Brandenburg in Bernau, Brandenburg Medical School (MHB) Theodor Fontane, 16321 Berlin, Germany; christian.butter@immanuelalbertinen.de; 13 University Medical Center Göttingen, Department of Cardiology and Pneumology, Georg-August University, 37075 Göttingen, Germany; andreas.schuster@med.uni-goettingen.de; 14 German Center for Cardiovascular Research (DZHK), Partner Site Göttingen, 10785 Göttingen, Germany; 15 Gastroenterology/Infectiology/Rheumatology, Charité University Medicine Berlin, 10117 Berlin, Germany; 16 German Centre for Cardiovascular Research (DZHK), Partner Site Berlin, 10785 Berlin, Germany

**Keywords:** acute myocardial infarction, myocardial area-at-risk, feature tracking, infarct size, myocardial salvage index, left ventricular recovery, strain

## Abstract

Background: Left ventricular global longitudinal strain (LV GLS) is a superior predictor of adverse cardiac events in patients with myocardial infarction and heart failure. We investigated the ability of morphological features of infarcted myocardium to detect acute left ventricular (LV) dysfunction and predict LV functional recovery after three months in patients with acute ST-segment elevation myocardial infarction (STEMI). Methods: Sixty-six STEMI patients were included in the C-reactive protein (CRP) apheresis in Acute Myocardial Infarction Study (CAMI-1). LV ejection fraction (LVEF), LV GLS, LV global circumferential strain (LV GCS), infarct size (IS), area-at-risk (AAR), and myocardial salvage index (MSI) were assessed by CMR 5 ± 3 days (baseline) and 12 ± 2 weeks after (follow-up) the diagnosis of first acute STEMI. Results: Significant changes in myocardial injury parameters were identified after 12 weeks of STEMI diagnosis. IS decreased from 23.59 ± 11.69% at baseline to 18.29 ± 8.32% at follow-up (*p* < 0.001). AAR and MVO also significantly reduced after 12 weeks. At baseline, there were reasonably moderate correlations between IS and LVEF (*r* = −0.479, *p* < 0.001), LV GLS (*r* = 0.441, *p* < 0.001) and LV GCS (*r* = 0.396, *p* = 0.001) as well as between AAR and LVEF (*r* = −0.430, *p* = 0.003), LV GLS (*r* = 0.501, *p* < 0.001) and weak with LV GCS (*r* = 0.342, *p* = 0.020). At follow-up, only MSI and change in LV GCS over time showed a weak but significant correlation (*r* = −0.347, *p* = 0.021). Patients with larger AAR at baseline improved more in LVEF (*p* = 0.019) and LV GLS (*p* = 0.020) but not in LV GCS. Conclusion: The CMR tissue characteristics of myocardial injury correlate with the magnitude of LV dysfunction during the acute stage of STEMI. AAR predicts improvement in LVEF and LV GLS, while MSI is a sensitive marker of LV GCS recovery at three months follow-up after STEMI.

## 1. Background

Despite impressive advances in diagnosis and management over the last decades, ST-segment myocardial infarction (STEMI) continues to be a major public health problem in the Western world and is becoming an increasingly important issue in developing countries [1,2]. Timely reperfusion with primary percutaneous coronary intervention (PPCI) within 12 h of symptoms onset is currently the treatment of choice in STEMI patients and has led to a significant improvement in outcomes and prognosis [3].

Left ventricular (LV) remodelling after STEMI can be induced by several factors, the first of which is the extent of myocardial injury [4,5]. Early reperfusion strategy and targeted medical treatment prevent changes in LV size, shape, and thickness involving both the infarcted and the non-infarcted LV segments and can influence LV function and prognosis [6,7].

Accumulating evidence suggests that markers of inflammation may be reliable indicators of the extent of myocardial injury and increased cardiovascular risk [8]. A recent pilot study in humans (CAMI1) demonstrated a significant correlation between C-reactive protein (CRP) concentration or CRP increase during the 32 h after the onset of STEMI symptoms and myocardial infarct size (IS) [9].

Although echocardiography remains the first-choice imaging modality in acute STEMI settings, cardiovascular magnetic resonance (CMR) imaging allows for a more detailed characterization of cardiac structure [10]. CMR provides a more accurate assessment of ventricular volumes, function, and myocardial mass and can detect a myocardial injury—oedema, necrosis/scar, haemorrhage, or microvascular obstruction [11,12]. Moreover, CMR-derived measures such as myocardial area-at-risk (AAR) or myocardial salvage index (MSI) have been proven as potential endpoints for clinical trials [13]. According to recent studies, LV strain estimated using the feature tracking (FT) technique is an important parameter that allows for the detection of more subtle functional changes in infarcted and remote myocardium [14,15]. LV global longitudinal strain (LV GLS) is a superior predictor of LV remodelling and adverse cardiovascular events [16]. Importantly, LV strain can be easily integrated with other CMR measurements in the diagnostic and prognostic algorithms.

The main objective of our study was to investigate whether the CMR-derived morphological changes of myocardial tissue during acute injury are associated with LV functional parameters and their change at three months follow-up after STEMI.

## 2. Methods and Materials

### 2.1. Study Design and Patient Population

This prospective, observational CMR study is a sub-study of CAMI-1 (CRP apheresis in Acute Myocardial Infarction study) trial (WHO International Clinical Trials Registry Platform: DRKS 00008988) [9]. Patients were consecutively enrolled into the study if they presented with first ST-segment elevation myocardial infarction (STEMI) and were treated with primary percutaneous coronary intervention (PPCI). The diagnosis of STEMI was based on typical symptoms, specific ECG changes (ST-segment elevation), increased troponin levels and detection of an occluded coronary artery during coronary angiography. Exclusion criteria were previous myocardial infarction (MI) or bypass surgery, cardiogenic shock, haemodialysis, acute infectious diseases, malignant or chronic inflammatory diseases, pregnancy or lactation period, contraindications for CMR imaging, the inability of follow-up CMR scans, and participation in another clinical trial. All patients were treated according to existing European Society of Cardiology (ESC) guidelines for the management of acute MI in patients presenting with ST-segment elevation and included PPCI, dual antiplatelet therapy, statin, beta-blockers and ACE inhibitors or ARB. [1]. Patients were enrolled at eight sites in Germany between 2015 and 2018 and underwent 2 CMR exams: at baseline and during follow-up. The first CMR was performed 2–9 days after STEMI diagnosis and PPCI, while the second CMR scan was scheduled and performed 12 ± 2 weeks after STEMI. The trial complied with the Declaration of Helsinki and was approved by national regulatory authorities and by the ethics committees of all participating centres. All patients provided written informed consent.

### 2.2. Cardiac Magnetic Resonance

CMR studies were performed with onsite available MRI scanners with two different field strengths: 1.5T (Siemens Avanto, Siemens Aera or Philips Achieva) and 3T (Siemens Skyra and Philips Signa HDxt). Cine images were acquired using a retrospectively gated balanced steady-state free precession (bSSFP) sequence with multiple breath-holds at end-expiration in three LV long-axis (two-chamber, three-chamber, and four-chamber) planes and a stack of short-axis slices covering the entire LV. The typical bSSFP sequences parameters were as follows: repetition time = 3.5 ms, echo time = 1.7 ms, flip angle = 60°, field of view = 420 × 420 mm^2^, reconstructed voxel size = 1.6 × 1.6 × 8.0 mm^3^, and 25–30 phases per cardiac cycle. T2-weighted images were obtained using fast-tau inversion-recovery fast spin-echo sequence in identical locations to LV short-axis cine images. Finally, a T1-weighted 3-dimensional (3D) contrast-enhanced inversion-recovery gradient-echo sequence was used for late gadolinium enhancement (LGE) image acquisition. Images were obtained 10 min after injection of 0.1 mmol/kg gadobutrol (Gadovist, Schering AG, Berlin, Germany).

### 2.3. Image Analysis

CMR studies were anonymized and analysed centrally in a blinded manner by experienced observers at MRI-Core-Lab (Department of Internal Medicine/Cardiology, German Heart Centre Berlin). Images were analysed offline using commercially available software (Medis Suite, version 3.1, Leiden, The Netherlands). LV volumes, LV myocardial mass, and LV ejection fraction (LVEF) were quantified according to the CMR consensus paper for LV function and mass quantification [17]. LV end-diastolic (LVEDV) and LV end-systolic (LVESV) volumes were estimated using manual planimetry of the endocardial and epicardial contours from the LV short-axis stack, and LVEF and LV myocardial mass were calculated. Papillary muscles were considered part of the blood pool and were not included in the LV myocardial mass. LV volumes and myocardial mass were adjusted to body surface area, which was determined using the Mosteller equation.

LV myocardial deformation analysis was performed using the CMR feature tracking (FT) technique. Three LV long-axis (two-chamber, three-chamber, four-chamber) and three LV short-axis (basal, mid-ventricular, and apical) cine images were uploaded into QStrain, version 2.0 module. LV endocardial and epicardial borders were contoured by a point-and-click approach at the LV end-diastolic phase. After the application of a tissue tracking algorithm, endocardial and epicardial borders were detected through all cardiac phases. If the contouring was inaccurate, borders were manually adjusted, and the tracking algorithm was repeated to ensure correct endocardial and epicardial surface delineation and precise myocardial strain measurements. The right ventricular upper septal insertion point was manually indicated to allow for accurate LV segmentation according to an AHA 16-segment model [18]. LV global longitudinal strain (LV GLS) was calculated by averaging the strain curves of three LV long-axis images, while LV global circumferential strain (LV GCS) was derived from three LV short-axis images.

The endocardial and epicardial contours drawn on cine images were transferred into T2-weighted and LGE images to estimate myocardial oedema, necrosis/scar area, and calculate area-at-risk (AAR), infarct size (IS), microvascular obstruction (MVO) size, and myocardial salvage index (MSI). The extent of myocardial oedema and myocardial necrosis/scar area was calculated using the signal threshold versus reference mean (STRM) > 3 standard deviations (SD) method as it provides the greatest accuracy with acceptable reproducibility compared to other signal intensity threshold techniques [19,20]. All algorithm-selected pixels in the myocardium were counted on each of the T2-weighted and LGE images. MVO was defined as a hypointense core in the areas of hyperenhancement on LGE images and was considered as part of the infarct zone. The following parameters were calculated as suggested by previous studies [13]:

AAR (%) = LV myocardial oedema/LV myocardial mass

IS (%) = LV necrosis/scar area mass/LV myocardial mass

MSI (%) = (AAR − IS)/AAR

### 2.4. Statistical Analysis

The statistical analysis was performed using Microsoft Excel and commercially available software (IBM SPSS Statistics version 26.0, Inc., Chicago, IL, USA and GraphPad Prism 8, San Diego, CA, USA). The Shapiro–Wilk test was used to determine whether the data were normally distributed. Body mass index (BMI), follow-up AAR, MVO, MSI, (∆) LV GLS, and LV GCS were not normally distributed. Continuous variables were expressed as mean ± standard deviation (SD) or median ± interquartile range (IQR) as appropriate depending on their distribution. Student‘s paired *t*-test or Wilcoxon test was used as appropriate to compare continuous variable differences between baseline and follow-up. Delta (∆) LVEF, LV GLS, and LV GCS were defined as changes from baseline to follow-up. Correlations between variables were tested with Pearson‘s product-moment correlation. Patients were further divided into groups based on the IS (<median IS and ≥median IS group), AAR (<median AAR and ≥median AAR group), and MSI (<median MSI and ≥median MSI group). The comparison of the groups was performed using a two-sided, independent-sample Student’s t-test for normally distributed data and the Mann–Whitney test for skewed data. Values of *p* < 0.05 were considered statistically significant.

## 3. Results

A total of 66 STEMI patients were finally included in the study. The mean age was 57.5 ± 10.1 years. The youngest patient was 36 years while the oldest was 80 years old. Study participants were predominantly male smokers. The mean time to PPCI was 4.5 h (4.5 ± 2.6 h). More than half of the patients had anterior STEMI, and the majority presented with Killip class 1 on admission. None of the participants had severe heart failure, classified as Killip class 3–4. More detailed clinical characteristics and demographics are shown in Table 1.

**Figure 1:**
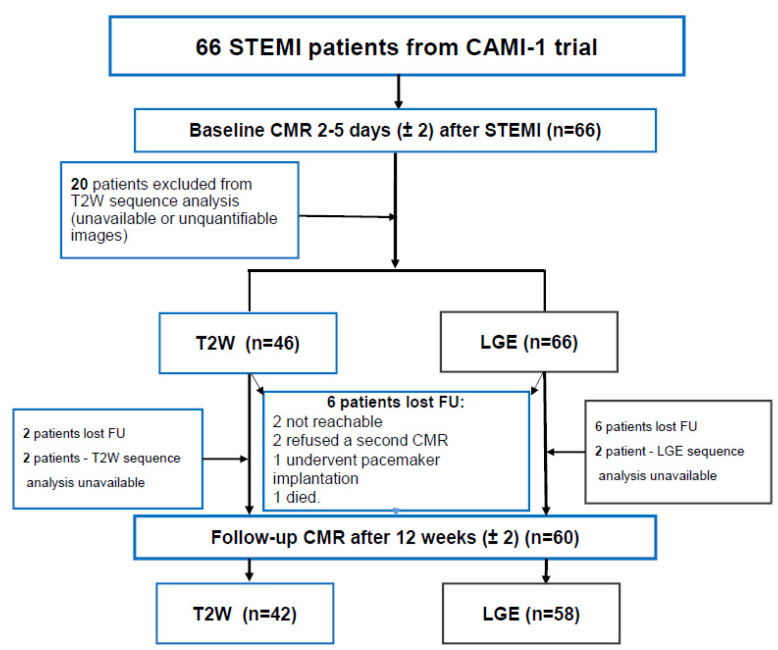
Study flowchart. Sixty-six STEMI patients were prospectively enrolled to have baseline and follow-up CMR. CAMI-1 = C-reactive protein apheresis in the Acute Myocardial Infarction study; CMR = cardiovascular magnetic resonance; FU = follow-up; LGE = late gadolinium enhancement; STEMI = ST-segment elevation myocardial infarction.

Six patients were lost during the follow-up: 1 patient died on day 114 after STEMI, 1 underwent pacemaker implantation and was excluded due to relative contraindication for a CMR scan, 2 patients refused to undergo a second CMR study, and 2 patients were unreachable (Figure 1).

After 12 weeks, there was no significant change in LVEDVi, while LVESVi was significantly reduced (*p* = 0.036) and LVEF improved (*p* < 0.001). The LVESVi was 41.30 ± 10.99 mL/m^2^ at baseline vs. 38.61 ± 13.78 mL/m^2^ at follow-up, while LVEF was 52.45 ± 7.07% at baseline vs. 55.78 ± 7.80% at follow-up. Systolic LV dysfunction (LVEF < 50%) was present in 25 (37.9%) patients at baseline and in 12 (18.2%) patients at follow-up. Severe LV dysfunction (LVEF < 35%) was observed in 1 (1.5%) patient and at both time points. There was a significant improvement in LV GLS at follow-up (20.18 ± 4.49 vs. −22.06 ± 5.39, *p* = 0.002) as well as in LV GCS (−25.68 ± 4.46 vs. −27.26 ± 6.37, *p* = 0.018).

MVO was present in 25 patients (37.9%) during the acute STEMI phase. Interestingly, 5 (7.6%) patients maintained hyperintense signal on T2-weighted images during follow-up, which might be considered residual myocardial oedema. Significant changes in myocardial injury parameters were identified after 12 weeks of STEMI diagnosis. IS decreased from 23.59 ± 11.69% at baseline to 18.29 ± 8.32% at follow-up (*p* < 0.001). AAR and MVO also significantly reduced after 12 weeks: AAR 37.19 ± 14.79% at baseline vs. 1.44 ± 7.08% at follow-up (*p* < 0.001) and MVO 1.04 ± 2.79% at baseline vs. 0.0 ± 0.0% at follow-up (*p* = 0.004). All functional and morphological CMR parameters at baseline and follow-up are listed in Table 2.

At baseline, there was a moderate negative correlation between IS and LVEF (*r* = −0.479, *p* < 0.001), while LV GLS (*r* = 0.441, *p* < 0.001) and LV GCS (*r* = 0.396, *p* = 0.001) were correlated positively with IS. AAR and LVEF (*r* = −0.430, *p* = 0.003) were also negatively correlated, whereas LV GLS (*r* = 0.501, *p* < 0.001) and LV GCS (*r* = 0.342, *p* = 0.020) showed a positive correlation with AAR.

The correlations between MSI and LV functional parameters were non-significant (*p* = 0.169 for LVEF, *p* = 0.575 for LV GLS, and *p* = 0.267 for LV GCS). At follow-up, only MSI and change in LV GCS over time showed a weak but significant correlation (*r* = −0.347, *p* = 0.021). More details are summarized in Table 3.

**Figure 2:**
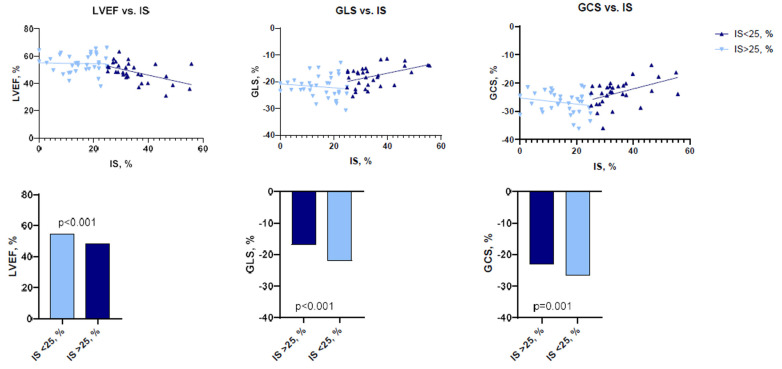
The impact of IS on LV functional parameters (LVEF, LV GLS, LV GCS) at baseline (*n* = 66). LV = left ventricular; GCS = global circumferential strain; GLS = global longitudinal strain; IS = infarct size; LVEF = left ventricular ejection fraction.

**Figure 3:**
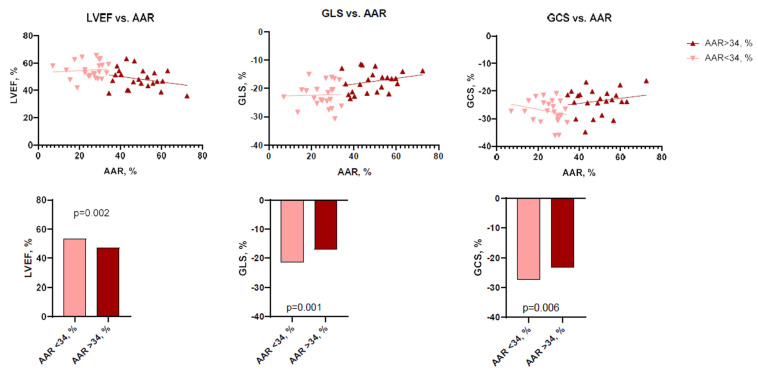
The impact of AAR on LV functional parameters (LVEF, LV GLS, LV GCS) at baseline (*n* = 46). AAR = area at risk; GCS = global circumferential strain; GLS = global longitudinal strain; LVEF = left ventricular ejection fraction.

**Figure 4:**
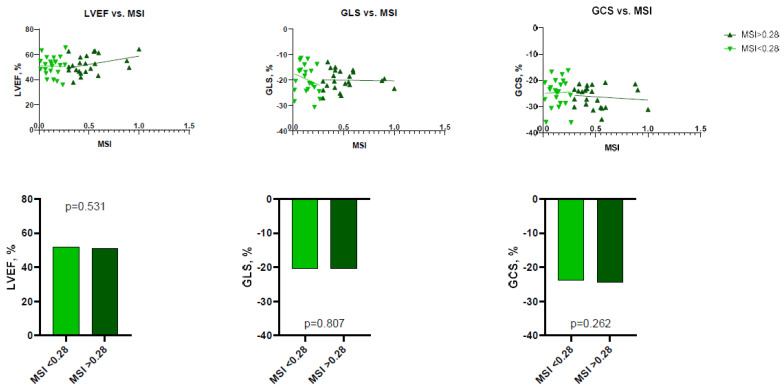
The impact of MSI on LV functional parameters (LVEF, LV GLS, LV GCS) at baseline (*n* = 46). GCS = global circumferential strain; GLS = global longitudinal strain; LVEF = left ventricular ejection fraction; MSI = myocardial salvage index.

**Figure 5:**
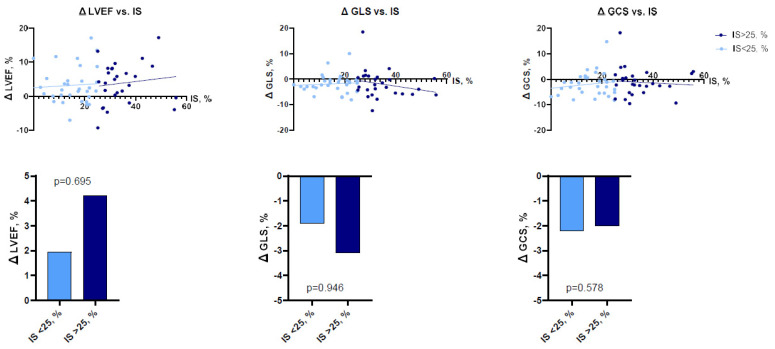
The impact of IS on LV functional parameters (LVEF, LV GLS, LV GCS) improvement (*n* = 59). GCS = global circumferential strain; GLS = global longitudinal strain; IS = infarct size; LVEF = left ventricular ejection fraction.

**Figure 6:**
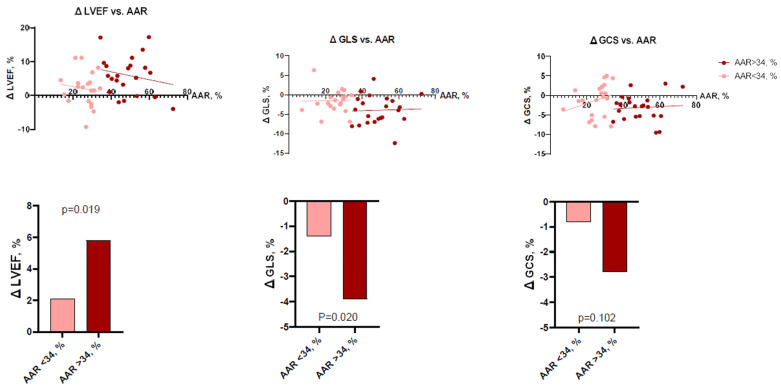
The impact of AAR on LV functional parameters (LVEF, LV GLS, LV GCS) improvement (*n* = 43). AAR = area at risk; ΔGCS = improvement in global circumferential strain; ΔGLS = improvement in global longitudinal strain; ΔLVEF = improvement in left ventricular ejection fraction.

**Figure 7:**
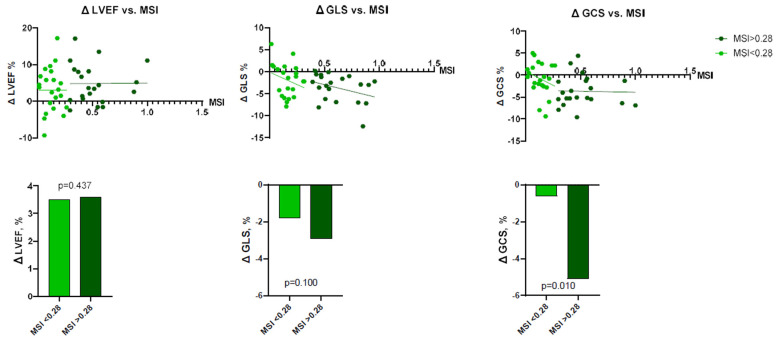
The impact of MSI on LV functional parameters (LVEF, LV GLS, LV GCS) improvement (*n* = 43). ΔGCS = improvement in global circumferential strain; ΔGLS = improvement in global longitudinal strain; ΔLVEF = improvement in left ventricular ejection fraction; MSI = myocardial salvage index.

To perform more detailed analysis, the data was divided into groups according to the median of IS (24.7%; IQR 14.3–32.0), AAR (34.3%; IQR 27.3–49.2), and MSI (0.28%; IQR 0.13–0.47). Analysis showed that patients with larger IS at baseline had lower LV functional parameters—LVEF, LV GLS, LV GCS, and vice versa (Figure 2 and Figure 3). There were no significant differences between the groups, according to MSI (Figure 4). On the other hand, it seems that IS at baseline does not influence the improvement of LV functional parameters (Figure 5). However, patients with larger AAR at baseline improved more in LVEF (*p* = 0.019) and LV GLS (*p* = 0.020) but not in LV GCS (Figure 6). In contrast, the patients with larger MSI at baseline significantly improved only in LV GCS (*p* = 0.008) (Figure 7). Interestingly, two different regression lines were observed for >25% and <25% IS, or >34% and <34% AAR, or >0.28 and <0.28 MSI, resulting in a kink in the curves in each individual comparison.

## 4. Discussion

The main purpose of our study was to determine whether the CMR features of tissue characterization (IS, AAR, MSI) are accurate predictors of LV functional recovery (LVEF and myocardial strain) after three months following acute STEMI.

Our study findings can be summarized as follows:

- AAR (quantified using T2-weighted images) and IS (quantified using LGE images) predict LVEF and LV GLS but not LV GCS improvement after STEMI;
- Acute myocardial injury characteristics quantified by CMR correlate with the extent of LV systolic dysfunction in the acute STEMI phase;
- MSI is associated with improvement in LV GCS after acute STEMI.

### 4.1. Myocardial Area-at-Risk

As described above, the myocardial area-at-risk was quantified using T2-weighted images in our study. We found that only a quantitative assessment of AAR was able to predict LV functional recovery (improvement in LVEF and LV GLS) after three months. Similar to a recent study of 50 subjects, there was a good agreement and correlation between AAR measurements, ECV, and T1 mapping values [21]. Despite a similar prognostic value, the T2-weighted imaging technique has two significant advantages compared to parametric mapping and LGE imaging. First, T2-weighted imaging does not require contrast administration; therefore, it is suitable for patients with severe renal dysfunction. Second, there is no need for a haematocrit value for AAR quantification, which can be underestimated due to excessive fluid therapy and haemodilution during acute STEMI.

Numerous studies have established an independent link between hyperglycaemia and adverse outcomes in patients with acute coronary syndromes. High blood glucose levels negatively influence coronary circulation and myocardial tissue through various pathophysiological mechanisms, such as cellular harm, programmed cell death, and compromised endothelial function [22]. Strict glycaemic control in STEMI patients may exert a cardioprotective effect through an anti-inflammatory mechanism and could be considered objective for future studies.

### 4.2. Quantification of Myocardial Scar Using Late Enhancement Imaging

More than two decades have passed since CMR imaging using the LGE technique enabled us to discriminate between irreversibly injured and viable myocardium [23]. In patients with previous myocardial infarction, LGE imaging allows us to detect and estimate the extent of myocardial scar tissue, which is considered an irreversible myocardial injury. The value of LGE assessment during the acute phase of myocardial infarction is less clear. Myocardial enhancement can be visible (detectable) within the first hours after myocardial injury and should be considered a sign of acute myocardial necrosis [24]. Previous studies have shown that IS predicts adverse LV remodelling, hospitalization due to heart failure during the first year after myocardial infarction, major adverse cardiac events (MACE), and all-cause mortality after STEMI [25,26,27]. We demonstrated that IS correlates with LV functional parameters (LVEF, LV GLS and LV GCS) at baseline. This has also been shown in previous studies [28]. We observed a significant decrease in LVEF and myocardial strain when the IS was exceeding 25% of LV mass. This finding is also in line with a similar study conducted by Larose et al., where an LGE extent ≥23% of LV during STEMI accurately predicted late LV dysfunction with a sensitivity of 89% and specificity of 74% [29]. To our knowledge, there is no described similar value for IS to predict LV dysfunction determined using myocardial tissue tracking (deformation imaging).

Interestingly, in the same study of 103 subjects with STEMI, authors found that myocardial injury quantified during the hyperacute phase using the LGE technique, predicts late LVEF recovery and adverse cardiovascular events [29]. However, in our study, we did not find a significant association between IS at baseline and improvement in LV systolic function after three months. This might be explained by a smaller study sample size (66 in our study vs. 103 in the study of Larose et al.) and earlier time point of the second CMR scan (3 months vs. 6 months), where a longer period could be necessary for a better LV functional recovery.

Novel CMR techniques, such as parametric mapping, might improve tissue characterization during acute myocardial injury. Pankaj et al. demonstrated that LGE imaging overestimates the IS during the acute phase of myocardial infarction compared with ECV fraction, which has been shown to have a superior agreement with IS estimated at follow-up scans [21]. According to previous studies, IS overestimation is a time-related issue, therefore, it is recommended to acquire LGE images approximately 15–30 min (not 10 min) after contrast injection or to perform a CMR scan on day seven after the cardiovascular event [30,31,32]. The quantification of IS using LGE remains essential for STEMI prognosis and risk stratification and is recognized as the primary endpoint in experimental, clinical trials, and cardioprotective studies. However, further validation studies, including accurate comparison with histological findings, are highly desirable [33,34].

### 4.3. Myocardial Salvage Index

MSI is a powerful marker of PPCI success and can be used as an endpoint in cardioprotective clinical studies [21]. The extent of MSI assessed by CMR predicts the outcomes in acute STEMI. It has a strong relationship with time from symptoms onset to PPCI, myocardial infarction localization (anterior STEMI), and TIMI flow grade before the PPCI procedure [35].

Our analysis demonstrated that MSI correlates better with the LV GCS change than LV GLS or LVEF. This is likely to be influenced by the larger extent of affected, oedematous, but non-infarcted myocardium that is expected to improve in systolic function after three months. The LV GLS reflects the shortening of subendocardial fibres and is first affected during myocardial ischemia with less ability to improve if myocardial necrosis occurs.

Our results support the previous research, showing that MSI is a reliable predictor of LV functional recovery and adverse LV remodelling [36,37]. In a study of 208 subjects Eitel et al. demonstrated that MSI is a powerful predictor of MACE and especially mortality compared with IS and MVO [26]. In addition, the MSI can be quantified using the T1 mapping technique and has been shown to be superior in predicting the recovery of LV function [21].

The ability of LVEF to stratify the risk of future cardiac events in patients with STEMI is limited. Previous studies showed that for a period of two years after STEMI, 67% of cardiac deaths occurred in patients with LVEF > 35%, while ventricular tachycardia was observed only in 8% of patients with reduced LVEF [37,38]. According to previous clinical trials, the incidence of SCD in acute MI with reduced LVEF varies between 8.5% at 2.5 years and 13.2% at 3.1% after hospital discharge [39,40].

Thus, despite primary and secondary prevention, high-risk patients remain unidentified. It is particularly important to include structural and functional (myocardial strain) CMR parameters in the routine clinical risk stratification model [41]. LV GLS is a more sensitive predictor of adverse prognosis at the one year follow-up after STEMI compared to LVEF and IS [26]. According to a recent study, LV GLS and LV GCS quantified by CMR-FT correlate significantly with LVEF, LVEDV, and IS [42]. Considering that the CMR-FT technique is less time-consuming, highly reproducible, has good to excellent intra-and inter-reproducibility, and the analysis does not appear to be influenced by the level of training, it may become the future gold standard for risk stratification in STEMI patients [43,44].

The individual risk assessment is the next step in personalized healthcare. The ability to predict adverse cardiovascular events using multiple CMR imaging biomarkers will significantly impact the decision-making process to achieve better care for patients with STEMI. Ongoing studies are focused on developing precision models using machine learning and will serve as a clinical decision support system to improve patient outcomes and assist healthcare providers [45].

## 5. Limitations

Our study is limited due to the small sample size, and a larger population could increase the strength of our research. Unfortunately, not all CMR scans were suitable for complete analysis, and not all patients underwent follow-up CMR. The parametric mapping was not performed in our cohort, as the mapping technique was unavailable in all participating centres. The inclusion of parametric mapping would certainly improve the prediction of LV functional recovery. We included only hemodynamically stable patients without significant comorbidities.

Moreover, the rate of advanced heart failure was relatively low; thus, our cohort cannot reflect the actual STEMI population. In addition, quantification of the IS may vary depending on the form, dosage, and timing of the contrasting agent after injection, as well as the timing after acute STEMI. Lastly, longer follow-up intervals might be beneficial in order to achieve complete LV recovery and remodelling.

## 6. Conclusions

The CMR tissue characteristics of myocardial injury (AAR, IS, and MSI) correlate with the LV dysfunction’s magnitude during the acute stage of STEMI. AAR predicts improvement in LVEF and LV GLS, while MSI is a sensitive marker of LV GCS recovery at three months follow-up after STEMI. As the reduction in CRP levels provides smaller infarcts and better cardiac function (LVEF, LV GCS, LV GLS), this could be considered an additional therapeutic option in STEMI.

## Figures and Tables

**Table 1:** Demographics and clinical characteristics of the study population.

Parameter	Value
Study population (number)	66
Age (years)	57.5 ± 10.1 (36–80)
Male sex	55 (83.3)
BMI (kg/m^2^)	27.4 ± 6.1
Smoking	51 (77.3)
Hypertension	37 (56.1)
Diabetes	13 (19.7)
Dyslipidemia	37 (56.1)
**Medical treatment before hospitalization**	
ACE inhibitor	6 (9.1)
ARB	11 (16.7)
Beta-blocker	10 (15.2)
Statin	5 (7.6)
Antiplatelet	5 (7.6)
**Basic clinical characteristics**	
Killip class 1	57 (86.4)
Killip class 2	9 (13.6)
Anterior STEMI	36 (54.5)
Time to PPCI (hours)	4.5 ± 2.6
**Number of affected coronary vessels**	
1	29 (43.9)
2	25 (37.9)
3	12 (18.2)

Baseline characteristics are reported as mean ± standard deviation or as absolute numbers with corresponding percentages. ACE = angiotensin-converting enzyme; ARB = angiotensin receptor blocker; BMI = body mass index; STEMI = ST-segment elevation myocardial infarction; PPCI = primary percutaneous coronary intervention.

**Table 2:** Comparison of functional and structural CMR parameters at baseline and follow-up.

Parameter	Day 1	Week 12	*p*-Value
LVEDVi, mL/m^2^	86.37 ± 15.58	85.75 ± 17.44	0.704
LVESVi, mL/m^2^	41.30 ± 10.99	38.61 ± 13.78	0.036
CI, L/min/m^2^	2.99 ± 0.64	2.83 ± 0.54	0.033
LVEF, %	52.45 ± 7.07	55.78 ± 7.80	<0.001
LV GLS (%)	−20.18 ± 4.49	−22.06 ± 5.39	0.002
LV GCS (%)	−25.68 ± 4.46	−27.26 ± 6.37	0.018
IS (%)	23.59 ± 11.69	18.29 ± 8.32	<0.001
AAR (%)	37.19 ± 14.79	1.44 ± 7.08	<0.001
MVO (%)	1.04 ± 2.79	0.0 ± 0.0	<0.001

Results are reported as mean ± standard deviation. AAR = area at risk; CI = cardiac index; GCS = global circumferential strain; GLS = global longitudinal strain; IS = infarct size; LVEF = left ventricular ejection fraction; LVEDVi = left ventricular end-diastolic volume index; LVESVi = left ventricular end-systolic volume index; MVO = microvascular obstruction.

**Table 3:** Correlation between myocardial injury characteristics and LV function.

	Infarct Size (*n* = 66)		Area at Risk (*n* = 46)		Myocardial Salvage index (*n* = 46)	
	*r*	*p*-Value	*r*	*p*-Value	*r*	*p*-Value
Baseline						
LVEF (%)	**−0.479**	<0.001	−0.433	0.003	0.206	0.169
LV GLS (%)	**0.441**	<0.001	0.501	<0.001	−0.085	0.575
LV GCS (%)	**0.396**	0.001	0.342	0.020	0.167	0.267
Improvement						
ΔLVEF (%)	0.118	0.371	0.236	0.124	0.116	0.452
ΔLV GLS (%)	−0.077	0.562	−0.269	0.081	−0.217	0.162
ΔLV GCS (%)	0.074	0.575	−0.124	0.421	−0.347	0.021

GCS = global circumferential strain; GLS mean = longitudinal strain; LVEF = left ventricular ejection fraction; Δ change from baseline to follow-up.

## Data Availability

Data are available upon request by the corresponding author.

## Abbreviations

AAR	Area at risk
CMR	Cardiovascular magnetic resonance
CRP	C-reactive protein
FT	Feature tracking
GCS	Global circumferential strain
GLS	Global longitudinal strain
IS	Infarct size
LGE	Late gadolinium enhancement
LV	Left ventricle/left ventricular
LVEF	Left ventricular ejection fraction
MI	Myocardial infarction
MSI	Myocardial salvage index
MVO	Microvascular obstruction
PPCI	Primary percutaneous coronary intervention
STEMI	ST-segment elevation myocardial infarction
